# Supplementary material for: Intrinsic bursts facilitate learning of Lévy flight movements in recurrent neural network models
Source: Sci Rep. 2022 Mar 23;12:4951. doi: 10.1038/s41598-022-08953-z (PMC8943163; doi:10.1038/s41598-022-08953-z)
Supplement: Supplementary file 1 — Supplementary Figures. [file 41598_2022_8953_MOESM1_ESM.pdf]

Supplementary materials
Intrinsic bursts facilitate learning of Lévy flight movements in recurrent neural network models

Morihiro Ohta, Toshitake Asabuki and Tomoki Fukai

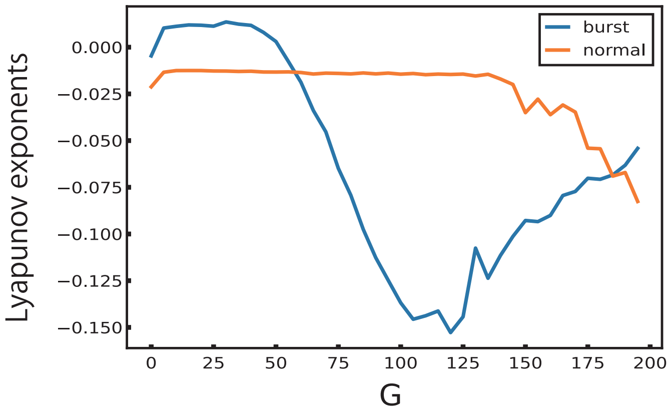

Supplementary Figure 1. The initial states of learning. The Lyapunov exponents of initial network states in the RS mode and bursting mode are shown for various values of G.

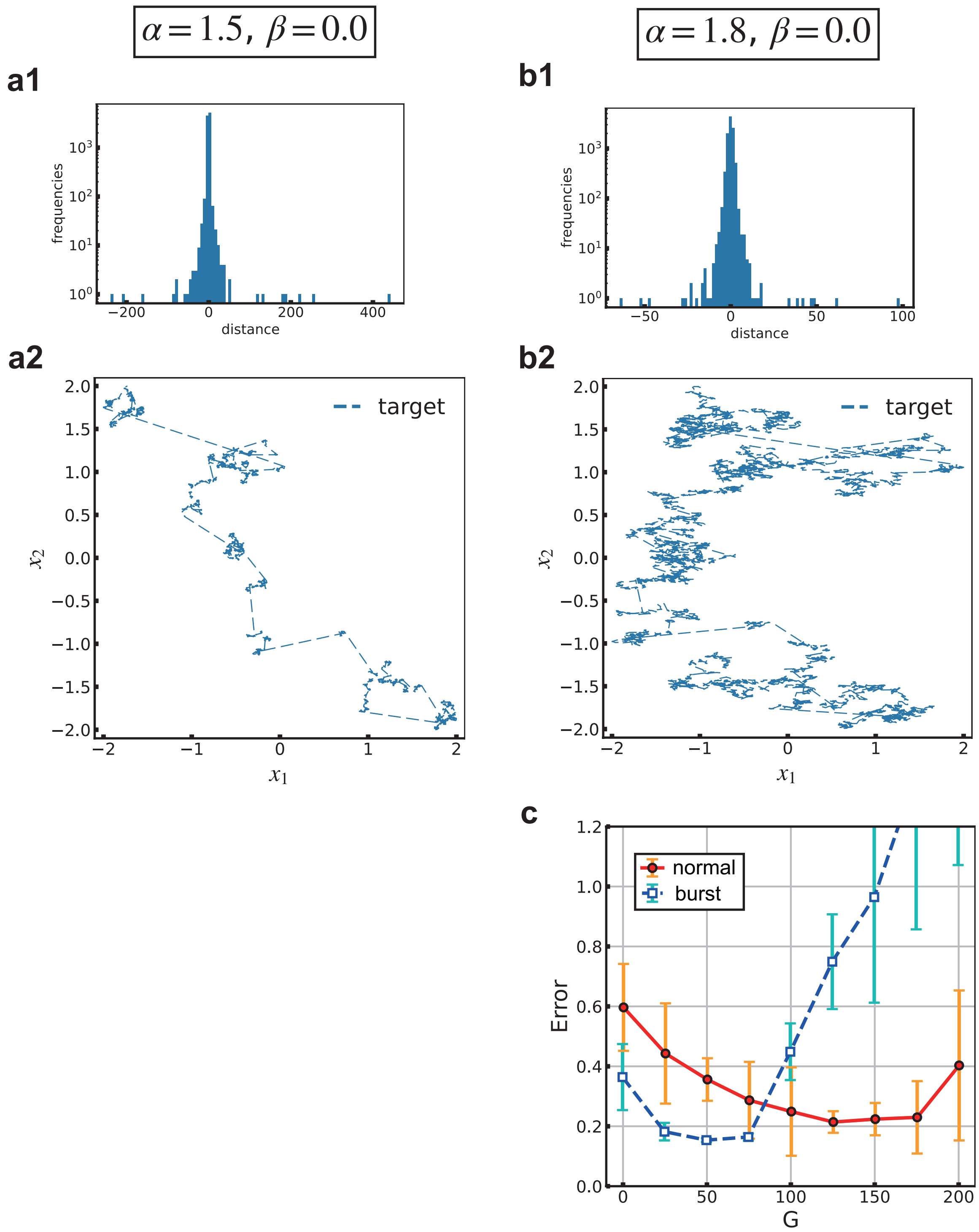

Supplementary Figure 2. Learning of a different type of Lévy walk. (a1, a2) The distribution of jump distances and the trajectory used in the main text are shown. The trajectory was copied from Fig. 1d. (a2, b2) Another distribution of jump distances and a typical trajectory generated with α =1.8 and β = 0.0. (c) Errors in learning were calculated for the target trajectory shown in (b2).

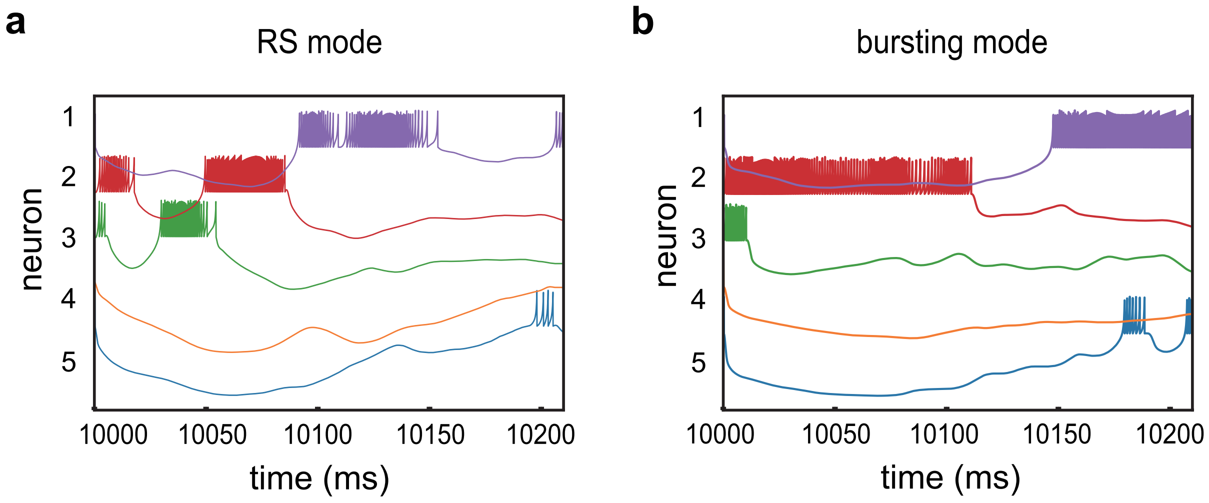

Supplementary Figure 3. Post-learning firing patterns. (a, b) Temporal spiking patterns after learning in the RS mode (a) or bursting mode (b) are plotted for five neurons. These patterns were obtained at the optimal coupling strengths of the

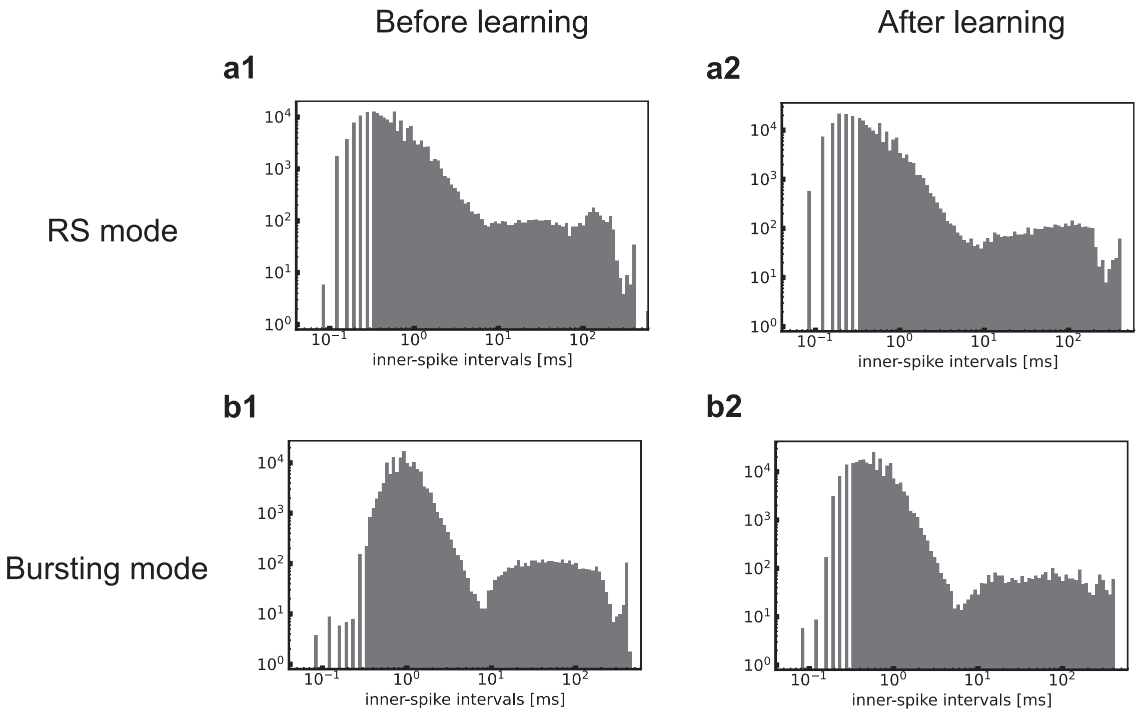

Supplementary Figure 4. The post-learning inter-spike-interval distributions. (a, b) The inter-spike-interval distributions are calculated over all neurons in the reservoir before (a1) and after (a2) learning in the RS mode (G = 170). Similar distributins are shown for the bursting mode (G = 50) before (b1) and after (b2) learning. The Izhikevich model used in this study does not take refractory periods into account and occasionally generates unrealistically short ISIs. Sharp upper bounds at 400 ms represent the length of the target signals used in the simulations.
